# Disparities in health related quality of life among Illinoisans diagnosed with depressive disorder: findings from the 2017 BRFSS

**DOI:** 10.1186/s12889-020-09041-5

**Published:** 2020-06-15

**Authors:** Kathryn Mazurek, James Ciesla, Rexford Akakpo

**Affiliations:** 1 Department of Health Sciences, College of Health and Human Sciences, Northern Illinois University, DeKalb, IL 60115 USA; 2 College of Health and Human Services, Bowling Green State University, Bowling Green, OH 43403 USA; 3 Department of Statistics and Actuarial Science, College of Liberal Arts and Sciences, Northern Illinois University, DeKalb, IL 60115 USA

**Keywords:** BRFSS, Depression, Mental health, HRQOL

## Abstract

**Background:**

The United States experienced severe mental health budget cuts in many states across the nation during the years of the largest recession since the Great Depression. Illinois had one of the hardest hit mental health budgets in the country. The massive mental health funding cuts in Illinois, combined with the state’s budget impasse, left fewer facilities available to provide treatment and support to those in need. Many of Illinois’s most vulnerable populations either had reduced access, or no access to care. Serious spillover effects were felt by emergency rooms, community hospitals, and the criminal justice system. Therefore, the purpose of this research is to examine disparities in Health Related Quality of Life for those with depression after the funding cuts in Illinois.

**Methods:**

Data from the 2017 Behavior Risk Factor Surveillance System was analyzed by using multivariate logistic regression models of the Health Related Quality of Life measures for Illinoisans diagnosed with depressive disorders.

**Results:**

According to the regression models in this study, disparities exist in HRQOL for Illinoisans with depressive disorders. In all of the HRQOL models, income was associated with a reduction in HRQOL. Additionally, disparities exist in HRQOL for certain age groups and those who are unemployed. Interestingly, the models did not show any racial disparities as anticipated.

**Conclusion:**

Without the basic policy-level deficiencies addressed, disparities in Health Related Quality of Life for Illinois’s most vulnerable populations will continue to exist as will costly economic spillover effects.

## Background

The United States experienced severe mental health budget cuts in many states across the nation during the years of the largest recession since the Great Depression [1, 2]. Illinois had one of the hardest hit mental health budgets in the country [3]. During years 2009–2013, Illinois cut 31.7% to mental health services resulting in roughly 187 million dollars cut from mental health initiatives [4]. Following the recession and starting in 2015 and lasting over 2 years, Illinois had the longest budget impasse in the history of the state and the history of the nation [5]. During both the recession and the budget impasse, many mental health facilities in Illinois were targeted in statewide cost-cutting plans [3]. Roughly 86% of mental health providers had to either reduce or eliminate services [6]. The closures and cutbacks left Illinois with fewer mental health practitioners, psychiatric facilities, and community mental health services [3, 7]. With fewer facilities available to provide treatment and support to those in need, many of Illinois’s most vulnerable populations either had reduced access or no access to care. Serious spillover effects were felt by emergency rooms, community hospitals, and the criminal justice system because these entities served as providers of last resort [3, 7]. The mental health situation became so dire that the popular press regularly wrote about the mental health crisis and national attention was drawn to Illinois when the Cook County Sheriff, Tom Dart, threatened to file a lawsuit against the State of Illinois [8]. Dart described Cook County Jail as “the state’s largest mental health provider” and said the county jail was overwhelmed with people whose offenses were more attributable to mental health issues than criminal impulses [8, 9].

The National Alliance for Mental Illness reported that 38.5% of Illinois residents have experienced poor mental health [10]. One group with poor mental health are those with depressive disorders (DD). According to the American Psychological Association, depression is the most expensive to treat among both mental health and substance abuse disorders and is the “sixth most costly health condition overall” [11]. Depressive disorders are common but serious disorders and are one of the leading causes of disability [12]. In Illinois, 15% of the population has been diagnosed with a depressive disorder and this translates to close to 2 million Illinoisans [13]. Depression occurs in all ages, races, and socioeconomic backgrounds [14]. However, many marginalized groups with depression have disproportionate differences in access to health care as well as service utilization which affects their health-related quality of life (HRQOL) [15]. HRQOL is measured by asking a series of questions about perceived physical health and mental health. Self-assessed HRQOL measures have become an important component of health surveillance and are generally considered valid indicators of service needs and intervention outcomes [12]. Self-assessed HRQOL are known to be a more powerful predictor of mortality and morbidity than many objective measures of health [16, 17].

The purpose of this observational study is to examine differences in HRQOL among those with depressive disorders living in Illinois. While the relationship between depression and the budget issues in Illinois is intuitive and commonly spoken about in professional settings and in lay literature, empirical evidence regarding the relationship has not been reported in the peer-reviewed academic literature. The findings will help contextualize the potential effects of Illinois mental health policy, budget cuts, and the budget impasse. The goal of this research is to make progress towards eliminating disparities among people with depressive disorders. Focusing on HRQOL will help health agencies bridge artificial distinctions between physical and mental health and will spur collaborations with a wider circle of health partners and stakeholders. Additionally, the results will provide insight and groundwork to facilitate recommendations for health policy, funding decisions, and public health intervention points.

## Methods

A secondary data analysis of the 2017 Behavior Risk Factor Surveillance System (BRFSS) was conducted to examine differences in HRQOL among those with depressive disorders living in Illinois. The BRFSS is a state-based system of health surveys that collects information on health risk behaviors, prevention practices, and health care access as it pertains to chronic disease [18]. Population based surveillance systems, such as the BRFSS, makes accumulating population data for states and communities a unique nationwide standard for identifying and tracking perceived unmet health needs and disparities.

The BRFSS telephone survey was conducted in collaboration with the Centers for Disease Control and Prevention (CDC) and the state health department to interview Illinoisans who are 18 years and older about risk factors, health behaviors and conditions that are “related to the leading causes of death” [19]. Interviews for the BRFSS are secured through a random digit dialing system known as The CATI system [19]. BRFSS study documentation including survey data, methodology, and design are publicly available [19].

This research used logistic regression modeling to identify the factors that contribute to disparities and to quantify the degree of any disparities that exist for those with depressive disorders. The models included the CDC healthy days core module, age, gender race/ethnicity, education level, marital status, employment status, health insurance status, and annual income. All participants were asked to identify the state in which they reside and if they have ever been told by a health care professional that they have a depressive disorder including depression, major depression, dysthymia, or minor depression. Only participants who self-reported that they live in Illinois and have been told that they have a depressive disorder were included in the analysis.

The instrument that is used in the BRFSS to assess HRQOL is the CDC’s Healthy Days Core Module (CDC HRQOL-4). The 4-item set of questions have been used in the BRFSS since the early 1990s [20]. The Healthy Days Core Module assesses an individual’s self-reported well-being through four questions about general health, physical health, mental health, and overall physical and mental health including activity limited days [20]. Self-reported general health status was dichotomized as excellent, very good, or good and fair or poor. The reference group used for general health status was excellent, very good, and good. The HRQOL questions regarding physical health, mental health, and poor physical and mental health including activity limited days, asked about how many days in the past 30 a participant had symptoms [20]. The number of days in the past 30 were dichotomized as 14 or more days compared with less than 14 days. Clinicians often use the cutoff of 14 or more days as an indication of clinical depression [21].

Differences were assessed by age categories (18–34, 35–49, 50–64, and 65+). Those who were 18–34 were the reference group. Gender was dichotomized and entered as male and female with male serving as the reference group. Also, self-reported primary race and ethnicity were non-Hispanic White, non-Hispanic African American, Hispanic, non-Hispanic other race only (including American Indian or Alaskan Native, Asian, native Hawaiian or other Pacific Islander), and non-Hispanic multiracial. Those who reported White non-Hispanic were the reference group. Level of education was entered into the models as did not graduate high school, graduated high school, attended college or technical school, graduated from college or technical school. Those participants who graduated college served as the reference group. Additionally, marital status was entered into the model and dichotomized as married or partnered and not married or partnered. Those who are not married or partnered included respondents who are divorced, widowed, separated, and never married. Employment status was entered into the model and dichotomized as employed for wages or self-employed and not employed which included respondents who were out of work, retired, students, homemakers, or those who were unable to work. A participant was considered to have health insurance if they responded yes to having any type of health insurance. Finally, household income levels were entered into the model as less than $ 25,000, $25,000 to less than $35,000, $35,000 to less than $50,000, and $50,000 and over.

BRFSS study documentation provides guidelines for complex sampling weights [19]. Therefore, weighting variables were entered into the models to account for the complex sampling design. Prevalence rates are reported in percentages and the reported N has been adjusted using the weighted analysis consistent with complex survey design. The analysis was performed using SAS 9.4. A *P* value of <.05 was considered significant.

## Results

As shown in Table 1, roughly 32% of the study population were younger than 35 years old, a about 16% were between 35 and 49, 17% were between the ages 50–64, and 35% were older than 65 years old. Over half, 64%, were female and 68% were non-Hispanic White, 12% were non-Hispanic Black and 13% were Hispanic with the remaining roughly 6% being non-Hispanic other race and 1% non-Hispanic multiracial. Approximately 14% of participants reported that they did not graduate high school, 28% graduated high school, 34% attended some college or technical school, and 24% graduated from college or technical school. Roughly 58% were married or partnered and about 47% of participants were unemployed at the time of the survey. Roughly 90% of respondents had some form of health insurance. About 17% of participants reported an annual income of less than $25,000, about 31% reported an annual income of $25,000–$34,999, 12% reported $35,000 to $49,999, and 40% reported an annual household income of $50,000 or more.

**Table 1:** *Prevalence of Self-Reported Depressive Disorders Among Illinoisans, 2017 Behavioral Risk Factor Surveillance System*

Characteristic	Frequency	%	Weighted Frequency
**Age group**			
18–34	181	32.1	562,404
35–49	123	16.3	285,209
50–64	162	17.0	299,264
65 or older	498	34.6	607,286
Total	964	100.0	1,754,163
**Gender**			
Male	325	35.8	62,816
Female	639	64.2	1,125,977
Total	964	100.0	1,754,163
**Race/Ethnicity**			
White only, Non-Hispanic	692	68.5	1,189,234
Black only, Non-Hispanic	113	11.8	204,970
Other race only, Non-Hispanic	33	6.0	103,613
Multiracial, Non-Hispanic	12	1.0	17,394
Hispanic	109	12.7	220,177
Total	959	100.0	1,735,388
**Level of education completed**			
Did not graduate High School	78	14.3	250,432
Graduated High School	234	27.4	479,974
Attended College or Technical School	289	34.2	599,983
Graduated from College or Technical School	363	24.1	423,773
Total	964	100.0	1,754,163
**Marital status**			
Married/Partnered	553	57.4	1,006,374
Not Married/Partnered	410	42.6	747,236
Total	963	100.0	1,753,610
**Employment Status**			
Employed	558	53.1	931,687
Unemployed	406	46.9	822,477
Total	964	100.0	1,754,163
**Health Insurance status**			
Insured	899	90.2	1,581,792
Uninsured	65	9.8	172,372
Total	964	100.0	1,754,163
**Income ($)**			
Less than $25,000	131	17.0	282,302
$25,000 to less than $35,000	273	30.9	512,164
**Characteristic**	**Frequency**	**%**	**Weighted Frequency**
$35,000 to less than $50,000	116	11.7	194,387
$50,000 or more	392	40.3	667,945
Total	912	100.0	1,656,798
**Health Status**			
Poor General Health	104	10.1	177,560
Poor Mental Health	309	33.8	587,563
Poor Physical Health	230	23.3	406,564
Poor Mental and Physical Health	190	23.1	350,765

### Health related quality of life

Table 2 displays the adjusted odds ratio (AOR) for respondents who reported their general health as fair or poor. Overall, individuals who were 50–64 were more likely to experience fair or poor general health as compared to the reference group. Also, those who were unemployed and all of those in the income categories that were less than $35,000 were significantly more likely to have poor general health than those who were employed or making $50,000 or more a year.

**Table 2:** *Self-Reported Poor General Health Status*

Characteristic	AOR	95% CI	
**Age**			
18–34	Reference	–	–
35–49	1.038	0.308	3.50
50–64	0.241*	0.092	0.632
65+	0.452	0.188	1.089
**Gender**			
Male	Reference	–	–
Female	1.543	0.885	2.691
**Race/Ethnicity**			
White only, Non-Hispanic	Reference	–	–
Black only, Non-Hispanic	1.911	0.860	4.244
Other race only, Non-Hispanic	1.499	0.188	11.950
Multiracial, Non-Hispanic	3.185	0.402	25.227
Hispanic	1.120	0.456	2.750
**Education Level**			
Did not graduate High School	0.969	0.312	3.006
Graduated High School	1.216	0.501	2.953
Attended College or Technical School	0.817	0.358	1.862
Graduated from College or Technical School	Reference	–	–
**Marital status**			
Married/Partnered	Reference	–	–
Not Married/Partnered	0.883	0.427	1.823
**Employment Status**			
Employed	Reference	–	–
Unemployed	0.193*	0.090	0.417
**Health Insurance status**			
Insured	Reference	–	–
Uninsured	0.530	0.206	1.363
**Income ($)**			
Less than $25,000	0.170*	0.066	0.438
$25,000 to less than $35,000	0.228*	0.101	0.513
$35,000 to less than $50,000	0.462	0.148	1.443
$50,000 or more	Reference	–	–

Notes: * A *P* value of <.05 is considered significant

Respondents who reported 14 or more bad mental health days which included stress, depression, and problems with emotions are shown in Table 3. Fourteen or more bad mental health days is a clinically significant cut off point for depression. Those individuals who identified as Hispanic reported being more likely than their White counterparts to experience poor mental health. The unemployed, unmarried, uninsured, and those making less than $35,000 were all more likely to report poor mental health as compared to those in the reference groups.

**Table 3:** *Self-Reported 14 or More Unhealthy Mental Health Days in the Previous 30 Days*

Characteristic	AOR	95% CI	
**Age**			
18–34	Reference	–	–
35–49	1.067	0.561	2.032
50–64	0.613	0.343	1.097
65+	1.309	0.774	2.212
**Gender**			
Male	Reference	–	–
Female	0.983	0.654	1.477
**Race/Ethnicity**			
White only, Non-Hispanic	Reference	–	–
Black only, Non-Hispanic	0.998	0.528	1.890
Other race only, Non-Hispanic	2.672	0.820	8.702
Multiracial, Non-Hispanic	1.061	0.182	6.188
Hispanic	2.088*	1.113	3.919
**Education Level**			
Did not graduate High School	1.111	0.494	2.497
Graduated High School	0.975	0.556	1.711
Attended College or Technical School	0.712	0.457	1.108
Graduated from College or Technical School	Reference	–	–
**Marital status**			
Married/Partnered	Reference	–	–
Not Married/Partnered	0.625*	0.419	0.932
**Employment Status**			
Employed	Reference	–	–
Unemployed	0.640*	0.411	0.996
**Health Insurance status**			
Insured	Reference	–	–
Uninsured	0.469*	0.238	0.921
**Income ($)**			
Less than $25,000	0.373*	0.194	0.717
$25,000 to less than $35,000	0.448*	0.265	0.756
$35,000 to less than $50,000	0.590	0.332	1.050
$50,000 or more	Reference	–	–

Notes: * A *P* value of <.05 was considered significant

Respondents who self-reported 14 or more days when their physical health was not good including physical illness or injury is shown in Table 4. Those between the ages of 50–64 and those 65 and older are more likely to report 14 or more days of poor physical health as compared to the reference group. Additionally, Hispanics were more likely to report poor physical health as compared to their White counterparts. Also, individuals who are not married or partnered, those who are uninsured, and those who are unemployed were more likely to report poor physical health. Participants with an annual household income of less than $50,000 were more likely to report poor physical health as compared to the reference group.

**Table 4:** *Self-Reported 14 or More Unhealthy Physical Health Days in the Previous 30 Days*

Characteristic	AOR	95% CI	
**Age**			
18–34	Reference	–	–
35–49	0.563	0.250	1.268
50–64	0.333*	0.156	0.711
65+	0.428*	0.214	0.856
**Gender**			
Male	Reference	–	–
Female	1.225	0.768	1.954
**Race/Ethnicity**			
White only, Non-Hispanic	Reference	–	–
Black only, Non-Hispanic	1.230	0.587	2.578
Other race only, Non-Hispanic	1.803	0.477	6.808
Multiracial, Non-Hispanic	2.028	0.348	11.809
Hispanic	2.856*	1.281	6.365
**Education Level**			
Did not graduate High School	0.464	0.184	1.172
Graduated High School	1.377	0.769	2.465
Attended College or Technical School	0.795	0.471	1.340
Graduated from College or Technical School	Reference	–	–
**Marital status**			
Married/Partnered	Reference	–	–
Not Married/Partnered	0.587*	0.370	0.933
**Employment Status**			
Employed	Reference	–	–
Unemployed	0.328*	0.189	0.569
**Health Insurance status**			
Insured	Reference	–	–
Uninsured	0.733*	0.279	1.927
**Income ($)**			
Less than $25,000	0.227*	0.106	0.490
$25,000 to less than $35,000	0.433*	0.241	0.780
$35,000 to less than $50,000	0.392*	0.201	0.765
$50,000 or more	Reference	–	–

Notes: * A *P* value of <.05 is considered significant

The last HRQOL measure which is shown in Table 5 asks respondents to report if their physical and mental health kept them from doing their usual activities, such as self-care, work, or recreational activities. Those individuals who were 50–64 were more likely to report poor physical and mental health as compared to the reference group. Additionally, those who were unemployed are more likely to report poor physical and mental health as compared to their employed counterparts. Individuals with an annual household income of less than $50,000 were also significantly more likely to have poor physical and mental health.

**Table 5:** *Self-Reported 14 or More Unhealthy Physical and Mental Days including Activity Limited Days in the Previous 30 Days*

Characteristic	AOR	95% CI	
**Age**			
18–34	Reference	–	–
35–49	1.305	0.520	3.276
50–64	0.289*	0.129	0.647
65+	0.672	0.316	1.429
**Gender**			
Male	Reference	–	–
Female	1.130	0.657	1.943
**Race/Ethnicity**			
White only, Non-Hispanic	Reference	–	–
Black only, Non-Hispanic	0.802	0.347	1.852
Other race only, Non-Hispanic	1.334	0.353	5.044
Multiracial, Non-Hispanic	1.645	0.236	11.458
Hispanic	1.734	0.770	3.906
**Education Level**			
Did not graduate High School	0.739	0.263	2.076
Graduated High School	1.254	0.650	2.416
Attended College or Technical School	0.902	0.512	1.591
Graduated from College or Technical School	Reference	–	–
**Marital status**			
Married/Partnered	Reference	–	–
Not Married/Partnered	0.657	0.401	1.076
**Employment Status**			
Employed	Reference	–	–
Unemployed	0.252*	0.132	0.479
**Health Insurance status**			
Insured	Reference	–	–
Uninsured	0.555	0.211	1.461
**Income ($)**			
Less than $25,000	0.279*	0.120	0.650
$25,000 to less than $35,000	0.336*	0.181	0.626
$35,000 to less than $50,000	0.447*	0.209	0.957
$50,000 or more	Reference	–	–

Notes: * A *P* value of <.05 is considered significant

## Discussion

According to the regression models in this study, disparities exist in HRQOL for Illinoisans with depressive disorders. In all of the HRQOL models, income was associated with a reduction in HRQOL. Our results are consistent with previously published research that those with lower income levels are more likely to have reduced HRQOL as compared to their high-income counterparts [22]. Given that many mental health services are unaffordable or cost prohibitive especially in Illinois where funding to mental health services was cut, it is logical that those individuals with lower incomes reported lower HRQOL states than their high-income counterparts. Additionally, in all of the models, those who are unemployed are more likely to report reduced HRQOL. Research suggests that higher risk of DD was associated with either being unemployed or out of the labor force [23]. Furthermore, literature has shown that high financial burden plays a role in reducing physical and mental HRQOL states [24]. Those individuals living below the poverty level are more likely to report psychological distress [25]. It is suspected that low income patients may not be able to spend time seeking or receiving mental health services because they need to seek out employment, work one or multiple jobs, take care of family members, and make difficult decisions regarding paying for either treatment or basic needs like housing or food.

The literature has shown that there is a relationship between age and HRQOL. The participants in this study who most often reported a significant decrease in HRQOL were those in the 50–64 age range. Research has shown that middle age is often associated with competing priorities such as taking care of both children as well as elderly parents. These competing priorities can be financially and emotionally taxing and may contribute to a reduction in health related quality of life [26]. The other age group in this study that reported a significant decrease in HRQOL were those in the 65+ age group. The Agency for Healthcare Quality and Research identified older adults as priority populations in healthcare and researchers found that these populations were less likely to be diagnosed or treated for mental health problems despite an ongoing need [27, 28]. Consideration must be given to older patient’s mental health concerns to ensure timely diagnosis and to mitigate further suffering. A reduction in HRQOL in both of these age groups highlights not only a missed opportunity for early intervention, but also the need for continued treatment.

Two of the HRQOL models in this study, the mental health model and the physical health model, show that those who were unmarried or not partnered reported a reduction in HRQOL as compared to their married counterparts. Several studies suggest a positive relationship between social support such as being married/partnered and HRQOL [29–31]. In other words, marriage or partnership has been shown to be a protective factor for health related quality of life. Our results do not show a significant difference in the models concerning aspects of mental health, but intuitively it makes sense that those who are partnered would have an advocate or someone who is able to intervene when there is a health care problem or concern.

Surprisingly, health insurance status was not significant in all of the models. The respondents in this study who did not have health insurance were not significantly more likely to have a reduction in HRQOL status with the exception of the mental health measure. This is interesting because patients without insurance are some of the most vulnerable as they have very few options for treatment. Research found that individuals who did not have any type of health insurance reported lower HRQOL status than those who have some type of health care insurance [32]. Mental health services for patients who are uninsured are often provided in short-term, acute care hospitals and emergency departments. There is anecdotal evidence that with the advent of the ACA, Illinois treatment providers were able to secure emergency treatment for those in serious need despite insurance status and despite the reduction or closures in overall treatment options.

Another interesting finding is that these models did not reflect racial disparities as anticipated. Research has shown that a higher proportion of African Americans have decreased HRQOL as compared to their White counterparts [33]. Research also suggests that the chronicity of DD is higher for African Americans and Hispanics than for Whites [34]. Furthermore, African Americans and Hispanics are more likely to rate their DD as severe or very severe and disabling [34, 35]. In this research, all HRQOL measures show that both African Americans and Hispanics with DD were not more likely than their White counterparts to report lower levels of HRQOL. One thought is that mental health is stigmatized in different racial and ethnic groups. In many of these groups, mental illness is thought to be overcome through willpower, heroism, and avoidance of morbid thoughts rather than by seeking mental health services [36]. In fact, African Americans often depend on social communities such as family and religious organizations for mental health support instead of visiting health care professionals [37]. Therefore, African Americans who participated in the study may never have been diagnosed by a health care provider as having depressive disorder or they may be unwilling to self-report having a depressive disorder. The same is true of those in Latino populations. Those in Latino populations place an exceptionally large priority on privacy and this may contribute to treatment barriers [38]. Latinos are often concerned about the stigma that comes with having mental health issues [36, 38]. Stigma is attached to being viewed as ‘loca’ and thus contributes to lack of appropriate treatment for depressive disorders [37]. The variations in racial and ethnic differences in stigma call for further consideration by Illinois treatment providers into the aspects of stigma that may be salient to particular populations.

### Limitations

The BRFSS is a cross sectional survey that relies on self-reports. Therefore, cause and effect relationships cannot be determined. Additionally, the questions that were included in the BRFSS do not include characteristics specific to mental illness. For example, age of onset, prescription medications and dosage, type of comorbidities, specialized mental health treatment, and remission or reoccurrence of the illness are important questions regarding mental illness. Despite this, the BRFSS gives both reliable and valid results as they relate to the HRQOL measures and is designed to provide broad based population information [39].

### Mental health policy and recommendations

Jails and emergency rooms are often the provider of last resort especially for the most vulnerable mental health populations. As is the case across the state of Illinois, those with mental illness often encounter the criminal justice system. Local jails are unable to meet the complex needs of those who are dealing with mental illness. In Illinois in 2010, the total cost to incarcerate an average daily population of roughly 45,500 was more than $1.7 billion and the cost per prisoner was about $38,300 annually [7]. The cost to provide appropriate mental health care for those who are incarcerated throughout the state would be staggering. Illinois has seen some success with mental health courts that provides long term treatment options to offenders as opposed to incarceration [40]. In 2015, 15 of Illinois’s 23 circuit courts reported implementing, or were planning to implement, mental health courts in their jurisdiction [40]. This is a step in the right direction for Illinois as it would be for many jurisdictions throughout the United States. Mental health courts have been found to be both beneficial to those who participate in the programs as well as the public at large with reduced recidivism rates and a reduction in cost to taxpayers.

The closure of mental health facilities strained hospitals throughout Illinois. Accordingly, reports have shown an increase of individuals seeking mental health treatment in emergency departments which in turn, led to patient hospitalization. The total number of hospital days in 2014 for Illinois adults hospitalized for depression was 136,374 days [7]. Furthermore, the average length of stay is much longer compared to hospitalization due to physical illness. Little progress has been made in reducing the length of stay for those who have been hospitalized because of a mental health diagnosis; this is despite a general absence of procedures or surgeries during a hospitalization for symptoms of serious mental illness [7]. Total hospital costs in Illinois for hospitalizations for depression in 2014 was $114,754,784 [7]. Total hospital costs in Illinois for hospitalizations for depression created a huge financial strain on the health care system while diverting money and other potential resources away from different treatment facilities and lower cost options. Going forward, Illinois must design, and consistently fund, a system that does not focus on the later stages of treatment or acute emergency treatment for those who are suffering from mental illness. Community mental health centers provide a plethora of services such as outpatient services, including specialized outpatient treatment for those who are chronically mentally ill. Community mental health clinics also provide 24 h-a-day emergency care, day treatment, partial hospitalization services, psychosocial rehabilitation, and screening for patients being considered for admission to mental health facilities [41]. Illinois must dedicate a larger amount of funding on these community-based clinics and programs and shift away from inpatient hospitalizations.

## Conclusion

Illinois struggles to recover from the fallout of an underfunded mental health system and serves as a cautionary tale as a recession in the U.S. looms. Despite public health efforts to reduce inequities, our findings show disparities in HRQOL exist for many of Illinois’s most disadvantaged residents. The funding and policy decisions most likely forced the most vulnerable Illinoisans to experience significant disease burden through unmet need and lack of treatment. Early intervention and appropriate treatment with continuing care will not only provide better treatment to patients but will also reduce the economic spillover effects such as those in emergency rooms, hospitals, and jails.

## Data Availability

The datasets analyzed are publicly available: https://www.cdc.gov/brfss/annual_data/annual_2017.html

## Abbreviations

ACA: Affordable Care Act
AOR: Adjusted Odds Ratios
BRFSS: Behavior Risk Factor Surveillance System
CDC: Centers for Disease Control and Prevention
HRQOL: Health Related Quality of Life
